# Influence of the shared epitope on the elicitation of experimental autoimmune arthritis biomarkers

**DOI:** 10.1371/journal.pone.0250177

**Published:** 2021-04-15

**Authors:** Anastasios Karydis, Indra Sandal, Jiwen Luo, Amanda Prislovsky, Amanda Gamboa, Edward F. Rosloniec, David D. Brand

**Affiliations:** 1 Department of Periodontology, University of Tennessee Health Science Center, Memphis, TN, United States of America; 2 Memphis VA Medical Center, Memphis, TN, United States of America; 3 Department of Medicine, University of Tennessee Health Science Center, Memphis, TN, United States of America; 4 Oregon State University, Corvallis, Oregon, United States of America; 5 Department of Microbiology Immunology and Biochemistry, University of Tennessee Health Science Center, Memphis, TN, United States of America; Universita Cattolica del Sacro Cuore, ITALY

## Abstract

Our previous studies have shown that inoculation of the oral cavity of “humanized” B6.DR1/4 mice with the periodontal pathogen *Porphyromonas gingivalis* results in an increase in the percentage of circulating Th17 cells, loss of bone and an exacerbation of experimental autoimmune arthritis. The aim of this study was to assess the role played by the human HLA-DRβ molecule containing the shared epitope supplied as a transgene to I-A˚ (murine class II null) C57BL/6 (B6) mice in driving these findings. We compared various immune response parameters as well as alveolar and peri-articular bone loss between humanized B6.DR1 (or B6.DR4) mice and their WT (B6) counterparts. We found that the presence of the shared epitope in the context of inoculation with *P*. *gingivalis* enhanced the percentage of Th17 cells generated, dramatically enhanced bone loss and importantly allowed for the generation of CCP2⁺ ACPAs that are not found in C57BL/6 or DBA/1 arthritic mouse serum. Due to the exceedingly complex nature of environmental factors impacting on genetic elements, it has been difficult to unravel mechanisms that drive autoimmune arthritis in susceptible individuals. The findings in this study may provide one small piece of this puzzle that can help us to better understand part of this complexity.

## Introduction

Rheumatoid Arthritis (RA) is a heterogeneous disease phenotype of autoimmune origin characterized by degeneration of the diarthrodial joints and accompanied by pain, swelling, loss of joint function and a severe decline in quality of life. For recent review, see [1]. At the tissue level, these changes are reflected by neutrophil influx, erythema, chronic inflammation, synovial proliferation, degradation of the cartilage and destruction of sub-chondral bone. The disease process involves both innate and adaptive immune responses, enlisting the activity T cells, B cells, antibody and the complement system.

Despite decades of research on the problem, questions about the etiology of RA remain. Numerous hypotheses have been proposed to explain the complex nature of the disease, but most believe that forces precipitating RA include a mixture of genetic, environmental and behavioral factors. It has been proposed that single factors alone are not capable of eliciting RA but that multiple “hits” might be necessary [2].

Among genetic predispositions for RA, perhaps the best known is the presence/absence of one of a limited set of MHC class II alleles known as the “shared epitope” (SE). The SE refers to amino acids 71–75 of the HLA-DRβ chain. To be included as a member of the SE family, the DRβ chain must bear the (Q/R)KRAA sequence. This class includes DRB1*0101, *0102, *0104; DRB1*0408, *0409, *0410, *0416, *0419; DRB1*1001 as well as DRB1*1402 and *1406.

A chief suspect among the environmental factors that influence arthritis are periodontal pathogens [3]. Periodontal Disease (PD) is a widespread pathology characterized by chronic inflammation in the oral mucosa that can result in loss of tooth function and damage to the underlying alveolar bone structure. While the etiology of PD is fairly well characterized, the exact prokaryotic members of the dysbiotic biofilm plaque communities that drive PD are not entirely clear. However, what is certain is that its expression has been linked to several other disease pathologies including (but not limited to) rheumatoid arthritis, diabetes, coronary artery disease and most recently Alzheimer’s (Alz) [4]. For a recent review of the effects of periodontal disease on systemic health, see [5]. While these clinical associations are well established, there remains a paucity of information concerning the mechanisms linking a dysbiotic oral microbiota with these disparate pathologies. However, one thing is for certain; genetic linkage studies clearly demonstrate that the SE is linked not only to RA, but also to PD [6] and Alz [7] as well.

Over the last couple of decades, rheumatologists have included anti-citrullinated peptide antibodies (ACPAs) in their panel of RA biomarkers. Serum measurement for these antibodies serve as standard of care because they have the capacity to predict the development of clinical pathology years before it is expressed, providing the physician time to apply prophylactic treatments to the patients to help slow the crippling disease progress.

ACPAs are specific for citrulline-containing host protein epitopes that are generated through the action of a class of peptidyl-arginine deiminase (PAD) enzymes that are upregulated under pro-inflammatory conditions. This PAD mediated post-translational modification of peptidyl-arginine on host proteins results in the formation of a citrulline-based neo-epitope towards which ACPAs are eventually produced.

While mammals express at least five isoforms of PAD enzymes [8], *P*. *gingivalis* is the only known prokaryote to possess its own (prokaryotic) peptidyl-arginine deiminase (pPAD) enzyme, capable of generating citrullinated neo-epitopes on host proteins as well as auto-citrullination of its own proteins. However, the pPAD enzyme exerts a synergistic activity because it works in concert with another signature enzyme, arginine gingipains (Rgp) which provides *P*. *gingivalis* with virulence in the form of enhancing tissue invasiveness, but may also play a key role in generating the tau tangles characteristic of Alz [4]. Rgp-mediated cleavage at arginine-X peptide bonds followed by pPAD-mediated citrullination of the resultant carboxy-terminal arginine results in citrullination of host proteins [2] as well as the subsequent generation of ACPAs directed against these neo-epitopes. The activity of these two enzymes and the resultant generation of ACPAs makes *P*. *gingivalis* an especially interesting subject in the search for autoimmune disease mechanism.

In order to take a mechanistic approach to elucidating the linkage between the SE, RA, PD, we chose to use a “humanized” mouse model in which I-A˚ (mouse class II-null) C57BL/6 mice were provided with a transgene encoding a chimeric form of mouse/human HLA-DRβ1. We call these mice B6.DR1 or B6.DR4 depending on the alleles expressed as transgenes in each line. Our recent studies [9] demonstrated that inoculation of the B6.DR1 mouse with the Gram-negative anaerobe *P*. *gingivalis* resulted in a rapid transient increase in the percentage of CD3⁺CD4⁺ PBMCs expressing the Th17 phenotype, the elaboration of proinflammatory cytokines and a dramatic systemic bone loss. We also showed that this treatment not only dramatically exacerbated an established autoimmune arthritis in this mouse, but could actually trigger a latent disease expression in arthritis-resistant mice as well as result in the elaboration of ACPAs that can be detected with CCP2.0 human diagnostic ELISAs.

In the present study, we used reporter-bearing WT C57BL/6 (B6) and both B6.DR1 and B6.DR4 mice in order to assess the role of the SE in altering some of these immune parameters. In these studies, we repeatedly inoculated the oral cavity of mice by brushing the gingival margins with a slurry of *P*. *gingivalis* mixed in carboxymethylcellulose (CMC) using a syringe delivery system developed in our laboratory. Multicolor flow cytometric analysis revealed that the percentage of PBMCs expressing the Th17 phenotype in B6.DR1 mice was significantly enhanced over that of B6. We also extended our previous findings by demonstrating that the significant bone losses we had observed in B6.DR1 mice with chronic oral infections could also be found in B6.DR4 mice. These bone losses were significant enough to be detected through visual examination of μCT reconstructions of the knee joints of the B6.DR4 mice.

Most importantly, we obtained serum from both B6 and B6.DR1 mice at baseline and also after type II collagen/CFA challenge for collagen induced arthritis (CIA) as well as in mice challenged only with *P*. *gingivalis*. These sera were applied to an Axis/Shield human ACPA diagnostic assay by substituting the supplied detection reagent with goat anti-mouse Ig 2˚ antibody. Despite the fact that both B6 and B6.DR1 strains made similar antibody responses to sonic extracts of *P*. *gingivalis*, we found that the presence of the DR1 transgene conferred the B6 mouse with the ability to make enhanced levels of ACPAs that were not possible in the WT mouse, either in the context of CIA disease expression or when inoculated with *P*. *gingivalis*.

## Materials and methods

### Animals

C57BL/6 mice expressing a chimeric mouse/human RA/PD susceptibility allele HLA-DRβ1(*0101) (or DRβ1(*0401)) as a transgene and containing a Foxp3^gfp^ reporter and an IL-17F^mrfp^ reporter were developed as described earlier [9] Mice are housed under SPF conditions and were carefully screened to assure the presence of all transgenes as well as the absence of murine class II in the B6.DR1 mouse. This study was carried out in strict accordance with the recommendations in the *Guide for the Care and Use of Laboratory Animals* of the National Institutes of Health. All work was performed under protocol 316941 which was approved by the Institutional Animal Care and Use Committee at the Memphis VA Medical Center. Numbers of animals for each experiment were determined after either power analysis or based on historical data where power analyses had failed to properly predict requirements. All anesthesia was performed according to our IACUC protocol using 1–2% isoflurane inhalant to effect. Animals were always observed after leaving the plane of anesthesia to make certain that they ambulated normally. No animals became severely ill during the study, no euthanasia prior to designed experimental endpoints was necessary, and no unanticipated adverse events occurred.

### Bacterial culture

*P*. *gingivalis* strain W83 was grown overnight in ATCC 2722 medium: tryptic soy broth supplemented with hemin (5mg/ml) and menadione (0.5mg/ml) at 37°C in a anaerobic chamber equilibrated with a mixture of 90% nitrogen, 5% carbon dioxide and 5% hydrogen. Bacterial cell counts were determined using a spectrophotometer with an optical density of 1.0 at 600nm corresponding to 1 x 10^9^ CFU/ml. 10^9^ CFU/ml of bacteria were harvested and washed three times in PBS, then re-suspended at 3.33 x 10^7^ /ml in PBS with 2% CMC.

### Bacterial inoculation

Briefly, 10–12 week-old B6.DR1, B6.DR4 or WT (B6) mice were put under brief isoflurane anesthesia, and 10^6^ CFU of live *P*. *gingivalis* in 30 μl of PBS with 2% CMC were administered from a customized tuberculin syringes to which coarse plastic bristles had been affixed in place of a needle. *P*. *gingivalis* was applied to the gingival margin of mouse maxillary molars daily for the periods of time indicated in individual experiments. In some experiments, a sham infected group received 30 μl of PBS with 2% CMC alone. Standard experiments included inoculations Tuesday through Friday for three consecutive weeks for a total of twelve inoculations. Other experiments included a chronic delivery for the periods indicated.

### Flow cytometry

At specific time points before, during and after inoculation of mice with live *P*. *gingivalis*, peripheral blood was drawn and stained with the following antibodies: Alexafluor 700 conjugated anti-mouse CD3ε, Peridinin Clorophyll/Cyanine 5.5 conjugated anti-mouse CD45, Pacific Blue conjugated anti mouse CD4 (all BD Biosciences) prior to acquisition on a SORP 5-Laser LSR II (BD Immunocytometry systems). Analysis was performed using FlowJo v10.5.

### μCT analysis

Mouse legs and heads were harvested and fixed in 2 changes of 10% neutral buffered formalin solution over 96 hours followed by a three-day gradient wash series ending in storage under 70%EtOH. They were then imaged in 70% EtOH using a Scanco μCT 40 at an 8 micron voxel size at 55 kV with 145μA over about 45 minutes as previously described [9].

### Detection of antibodies to *P*. *gingivalis*

Solid-Phase ELISA was performed using a modification of the methods described in Van Tilburg *et al*. [10], briefly, log-phase cultures of *Porphyromonas gingivalis* were sonicated for 20 minutes at 20% power on a Branson Digital Sonifier Model 450, using a stirred ice bath to dissipate heat from the sonication. The extract was centrifuged to remove insoluble particulates and used for coating the wells of U-vinyl microtiter plates. Plates were washed and blocked with bovine serum albumin and stored at 4˚C prior to use. Serum from five different time points, both before and after inoculations with *P*. *gingivalis* was collected from a minimum of four each B6 and B6.DR1 mice and stored at -20˚C prior to use in ELISA. Serum from B6 and B6.DR1 mice inoculated with CMC was used as a control. Standard curves were generated using affinity purified antibody isolated from hyperimmune serum of *P*. *gingivalis*-challenged mice. An example standard curve is provided as S1 Fig. Serum was diluted to 1:1000 for the primary ELISA and then subsequent ELISAs were generated with 2-fold serial dilutions of serum samples ranging from 1:1,000 to 1:128,000. Plates were incubated overnight, washed and then incubated for 2 hours with HRP-labeled goat anti-mouse IgG1 (Southern Biotech #1071–05) at a 1:10,000 dilution. Plates were washed again, developed with o-Phenylemenediamine, the reaction stopped and absorbance was measured on at 490nm with a background of 650nm subtracted.

### Detection of antibodies to citrullinated protein antigens

Serum samples were obtained at the times indicated from naïve mice, those challenged for collagen induced arthritis following clinical signs of disease arthritic mice and those mice inoculated with *P*. *gingivalis* alone. A minimum of five mice per group was used in each analysis. These sera were subjected to a commercial (Axis/Shield, Scotland, Cat. #FCCP600) ELISA according to the manufacturer’s instructions, including the selection of the appropriate mouse secondary detection reagent, a horse radish peroxidase-labeled goat anti-mouse IgG2b (LifeTechnologies catalog number M32407).

### Collagen induced arthritis

Arthritis was induced in B6.DR1 mice following our well established protocol [11]. A minimum of ten mice per group of B6 and B6.DR1 mice were challenged with 100 μg of native chicken type II collagen (extracted and purified in our laboratory) emulsified in CFA made from 15% (v/v) mannide monooleate in heavy mineral oil. Mice were visually assessed for the appearance of clinical signs of arthritis according to protocol [11].

## Results

### T cell phenotype

**Fig 1:**
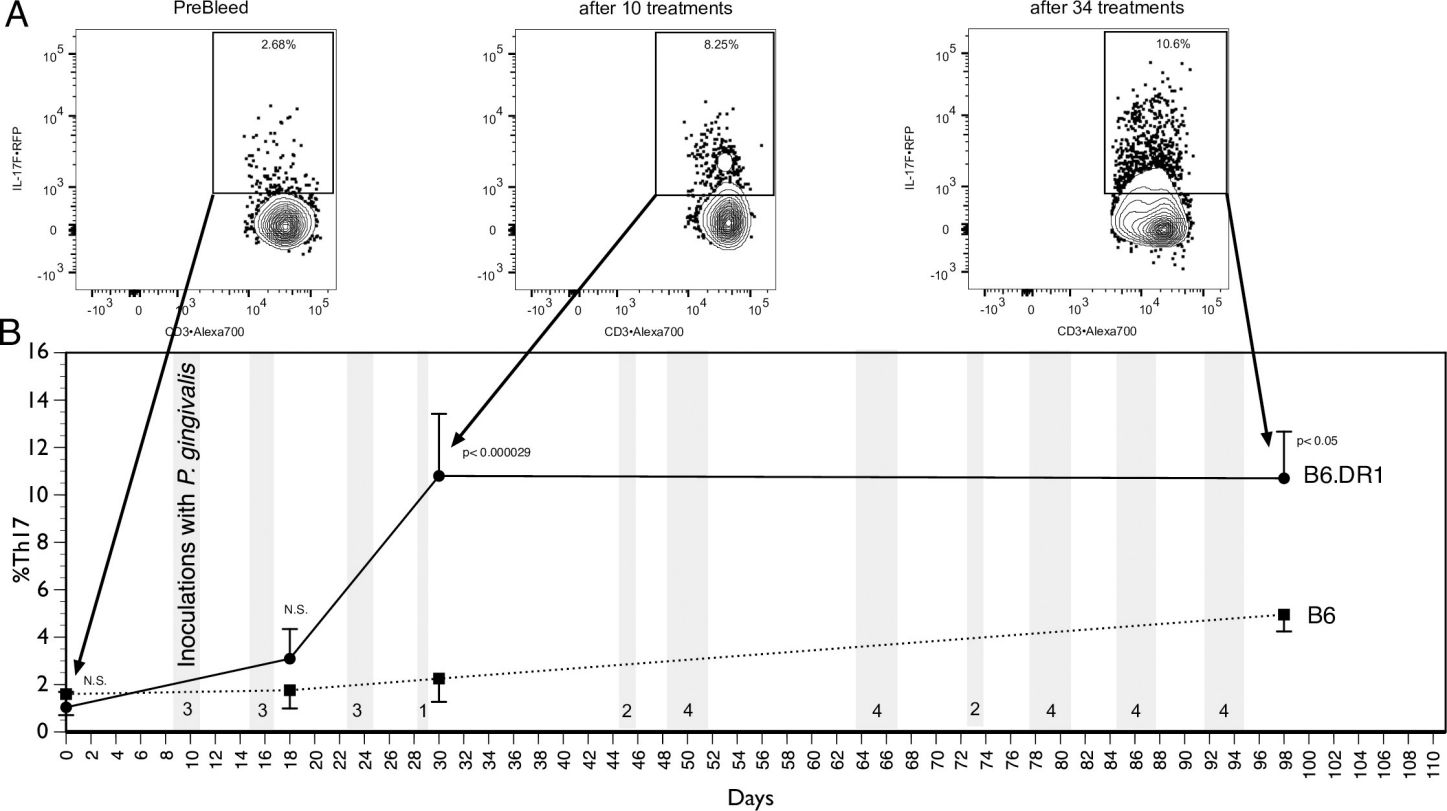
An elevated percentage of CD4⁺ T cells in peripheral blood that express the Th17 phenotype are found in SE-expressing mice. Representative multicolor flow cytometric scatter plots before (A) and after (B) inoculation of B6.DR1 mice with *P*. *gingivalis*. Square gates represent the percentage of DAPI⁺CD45⁺CD3⁺CD4⁺ cells among the PBMCs that are expressing IL-17F. Peripheral blood samples from WT (B6) or B6.DR1 mice were analyzed by flow cytometry (C) before (Day 0) and at three times post oral inoculation (vertical grey bars) with *P*. *gingivalis*. Events are gated on DAPI⁺CD45⁺CD3⁺CD4⁺ cells, and results are expressed as the percentage of these cells among PBMCs that are of the Th17 phenotype. Stats: t-test.

In order to understand the role of the SE in the development of the immune response to chronic PD, we inoculated both B6 and B6.DR1 mice to *P*. *gingivalis* thirty-four times over the course of ninety-five days (Fig 1). Using their built-in reporter constructs, we used multicolor flow cytometry to measure the percentage of circulating CD4⁺ T cells expressing the Th17 phenotype. While our previous studies had already demonstrated that the B6.DR1 mice generated a substantial shift in the profile of circulating CD4⁺ T cells expressing the Th17 phenotype, this study indicated that the wild-type (B6) mice did not exhibit this robust response but instead showed a mild increase in the percentage of CD4⁺ T cells expressing the Th17 phenotype. Differences in responses to vehicle (CMC) challenge were not found (S2 Fig).

### Anti-*P*. *gingivalis* antibody production

**Fig 2:**
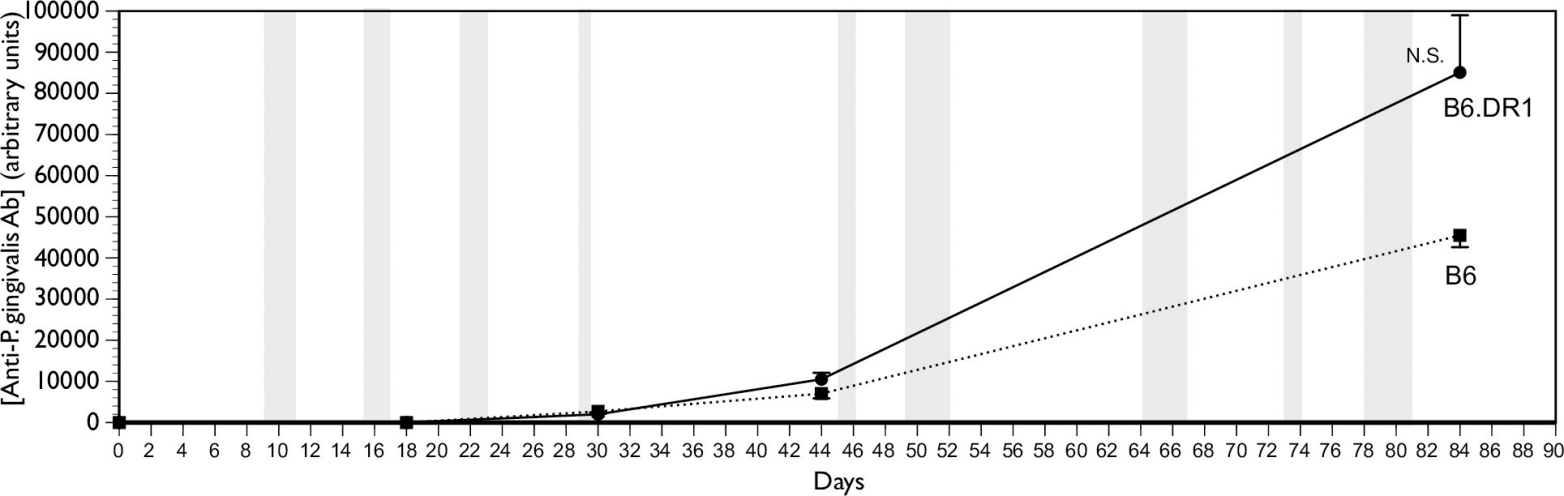
Both B6 and B6.DR1 make antibodies to *P*. *gingivalis*. Serum samples from both B6 and B6.DR1 mice taken at time points both before and after several rounds of inoculation with *P*. *gingivalis* reveal that both strains produce antibody to the pathogen at similar titers. NS = not statistically significant.

The central function of the adaptive immune system is to maintain homeostasis through an exquisitely specific selective defense system that has adapted to destroy invading pathogens such as *P*. *gingivalis*. One of the primary ways through which this is achieved is through the elaboration of antibodies specific for the pathogen that can be used to opsonize it and aid in its eventual clearance. We had measured an enhanced Th17 response in the peripheral blood of mice bearing the HLA-DR1β transgene relative to that of wild-type B6 mice. We used an ELISA to analyze the serum for the presence of antibodies that would bind to sonic extracts of heat-killed preparation of the same *P*. *gingivalis* organisms that were used to inoculate the mice. We found (Fig 2) that both WT (B6) and B6.DR1 mice made antibodies to sonic extracts of *P*. *gingivalis* and the differences in the levels were not statistically different from one another. Serum samples taken at identical time points from four B6 and four B6.DR1 mice inoculated with CMC did not result in any measurable antibody (not shown).

### ACPA production

**Fig 3:**
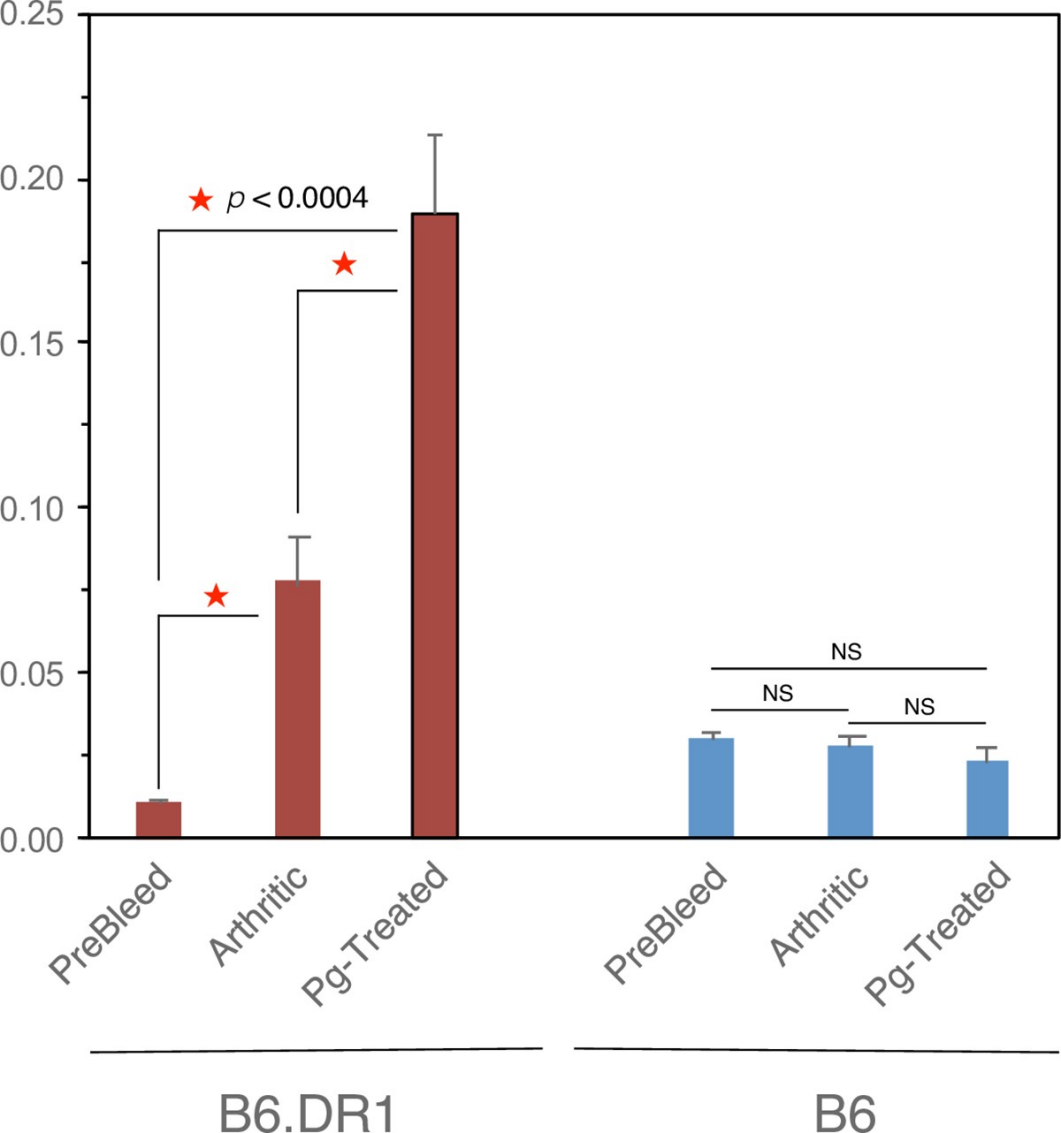
The presence of the shared epitope drives an enhanced production of ACPAs as a result of autoimmune arthritis induction or after inoculation with *P*. *gingivalis*. Serum from B6.DR1 or WT B6 mice was applied to commercial diagnostic ACPA ELISA by substitution of the 2˚ antibody as described in the *Materials and Methods* section. Histograms indicate ACPA levels in serum from mice subjected to either collagen induced arthritis experiments (Arthritic) or those repeatedly inoculated with *P*. *gingivalis* as indicated. *p<0.0004 by t test.

In addition to analyzing the serum from B6 and B6.DR1 mice for the presence of antibodies to *P*. *gingivalis*, we used a commercial diagnostic system to measure the relative quantities of antibodies to citrullinated peptides (ACPAs). In order to do this, we followed the suggestions of the manufacturer (Axis/Shield) and substituted the supplied anti-human IgG detection antibody with a goat anti-mouse Ig instead. We found (Fig 3) that ten inoculations of B6.DR1 mice to *P*. *gingivalis* over the course of 16 days resulted in expression of ACPAs whereas identical treatment of wild-type B6 mice produced almost no detectable ACPAs at all. There were no ACPAs detected in either B6.DR1 or WT (B6) mice following 24 inoculations with CMC over the course of eight weeks.

As with our previous analyses of DBA/1 mouse serum (not shown) no ACPAs were detected in the serum of WT (B6) mice even in the context of a severe arthritis score.

### Arthritis

**Fig 4:**
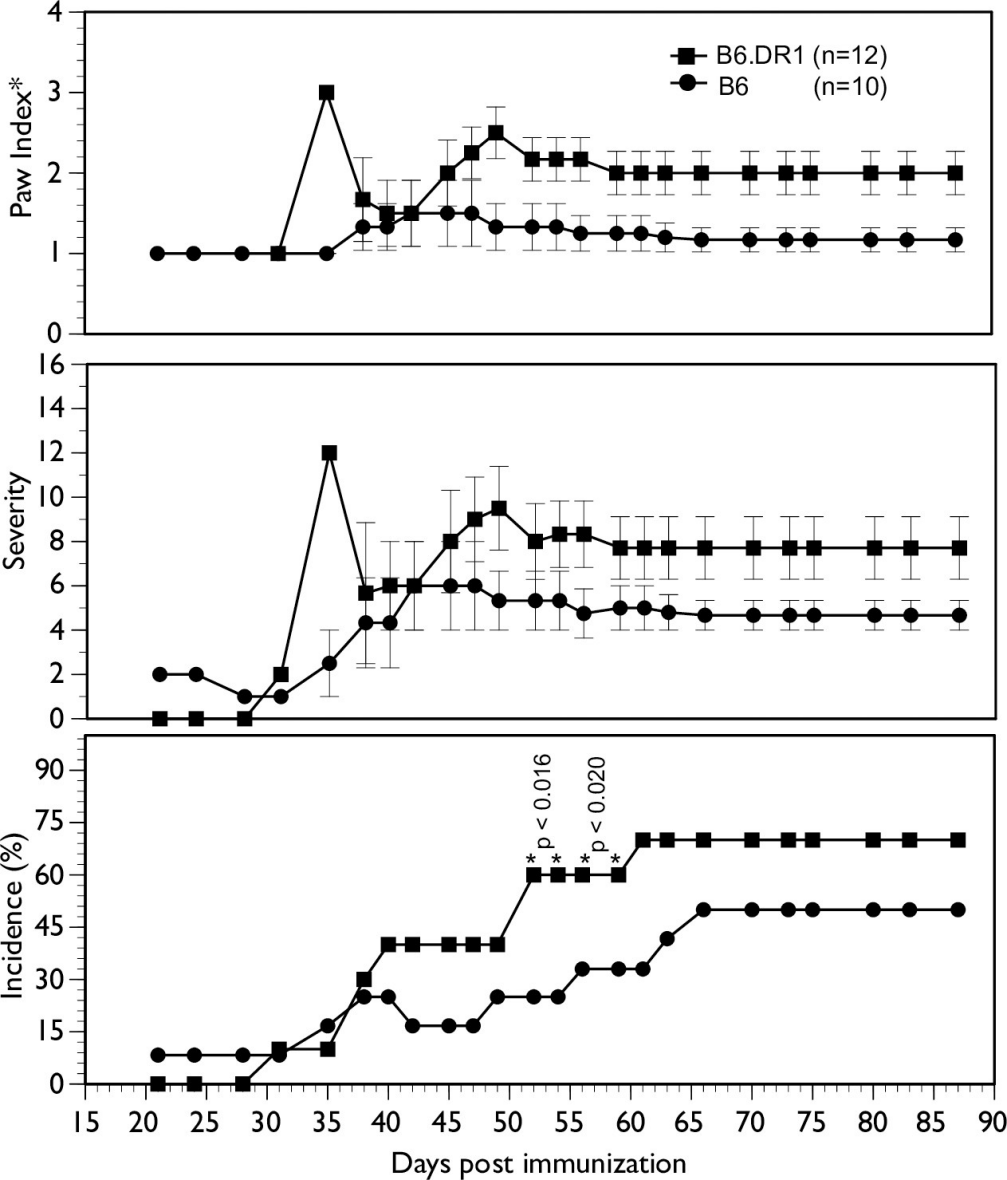
Collagen-induced arthritis is only marginally worse (if at all) in B6.DR1 mice. B6.DR1 or B6 mice were challenged with chicken type II collagen emulsified in CFA as described in the *Materials and Methods* section. While chi square analyses show statistical differences in the incidence of mid-course arthritis, differences in severity (average score per arthritic mouse) and paw index (average number of arthritic limbs per arthritic mouse) exhibited only slight differences between strains. Stats: chi-square analysis.

In order to assess the effect that the SE may play on the development of arthritis, we challenged both WT (B6) mice and B6.DR1 mice with chicken type II collagen emulsified in Complete Freund’s Adjuvant using our standard CIA protocol [11]. While the challenge was somewhat unimpressive in the overall final incidence (70% in B6.DR1 and 50% in B6 mice, the onset and kinetics of disease were unremarkable (Fig 4)). Notably, there was not a great deal of difference between the incidence and severity of disease between those mice bearing the SE and those with a WT genotype. The results only reached statistical significance for incidence during mid-course (between days 50 and 60) and then only transiently. Note also that the CIA depicted in Fig 4 and the ACPAs in the serum from arthritic mice depicted in Fig 3 represent the only CIA used in this study. All of the bone loss described below is a result of the action of inoculations with *P*. *gingivalis* only.

### Bone loss

**Fig 5:**
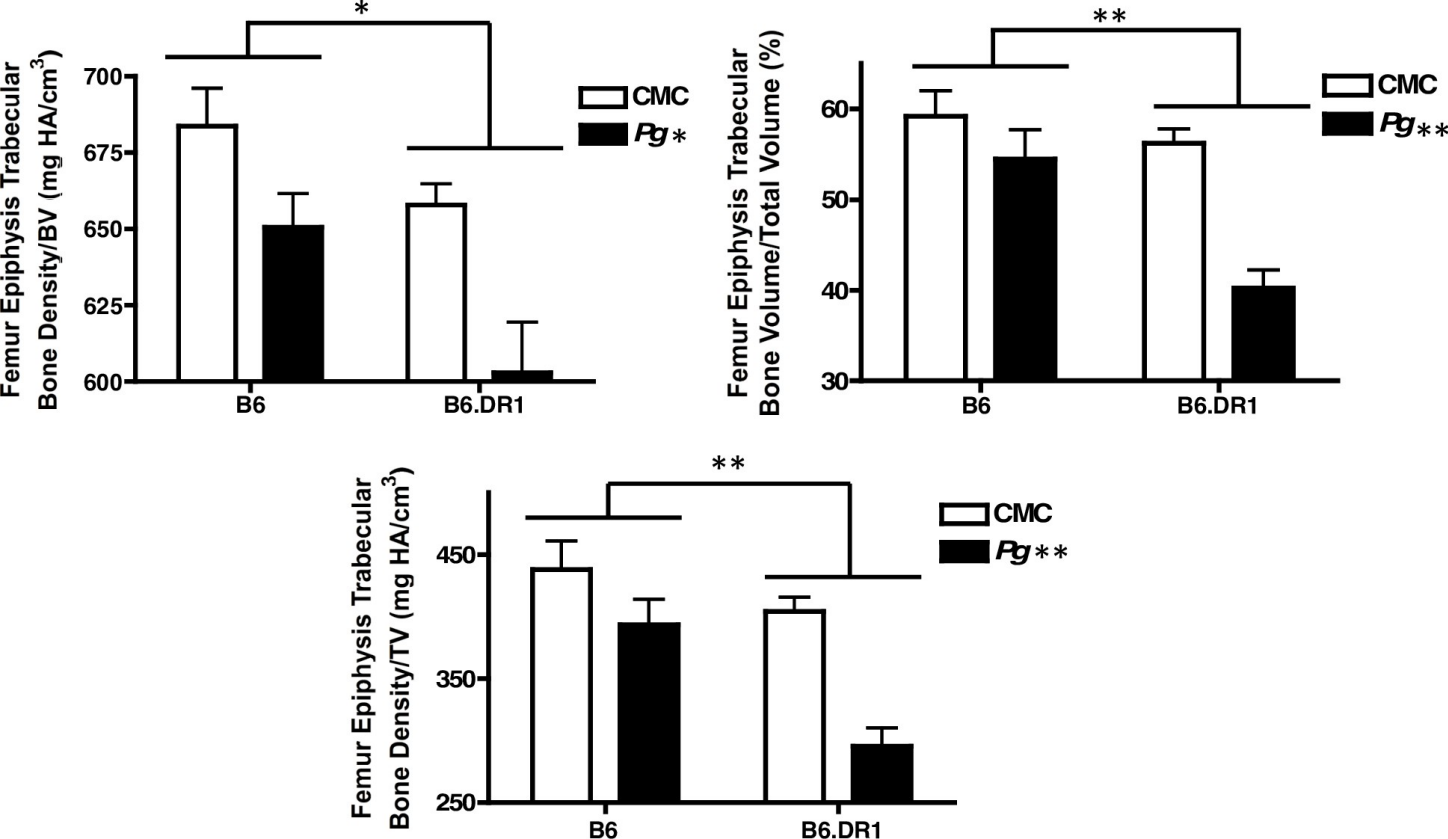
Trabecular bone loss is greater in DR1-bearing mice. B6 or B6.DR1 mice were repeatedly inoculated with *P*. *gingivalis* or vehicle (carboxymethyl cellulose–CMC) as described in the *Materials and methods* section. Following sacrifice, whole legs were subjected to μCT analysis. * p<0.05 by strain and by B6.DR1 treatment, ** p<0.01 by strain and by B6.DR1 treatment. Stats: t test.

**Fig 6:**
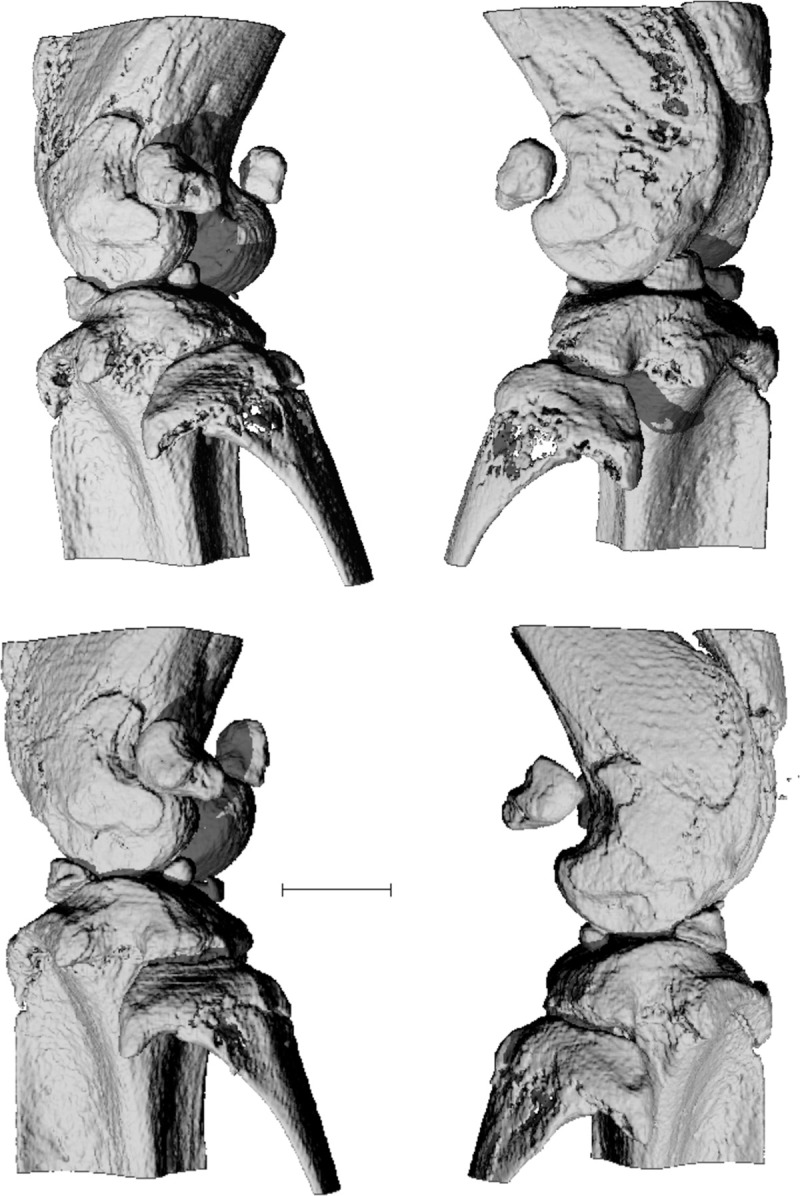
*P*. *gingivalis* mediated loss of trabecular bone can be seen in three-dimensional μCT reconstructions of knee joints from DR4-bearing mice. B6.DR4 mice were repeatedly inoculated with *P*. *gingivalis* while control mice were left untouched as described in the *Materials and Methods* section. Following sacrifice, whole legs were subjected to μCT analysis. Bone loss is especially apparent in fibula head for both the left knee (see also S3 Fig) and the right knee (see also S4 Fig) (top row) of a mouse treated with *P*. *gingivalis* relative to a left knee (see also S5 Fig) and a right knee (bottom row) from control B6.DR4 mice. These data are more easily visualized in animations made from the knees above that are provided as supplemental files. bar = 1mm.

Our previous work indicated that all of the immune responses to chronic inoculation with *P*. *gingivalis* that we measured in B6.DR1 mice resulted in significant loss of both alveolar bone and peri-articular bone. In this study, we compared these responses to those of WT (B6) mice given the same treatments, as well as to B6.DR4 mice. We found that the presence of the SE conferred an enhanced bone loss to the mice treated with *P*. *gingivalis*. Despite previous studies demonstrating that B6 mice are resistant to bone loss driven by *P*. *gingivalis*, our studies demonstrated that repeated inoculation with *P*. *gingivalis* resulted in a significant volume of peri-articular bone loss, even in the absence of the SE. However, Fig 5 clearly demonstrates that the presence of the RA susceptibility allele can drive an enhancement (over levels seen in B6 mice) in loss of trabecular bone, even at sites far-removed from the oral cavity where the inoculation takes place. We repeated the analysis of bone loss using C57BL/6 mice bearing a different RA/Periodontal Disease susceptibility allele HLA-DR4. μCT analysis revealed that the average bone mineral density for the knee joints of B6.DR4 mice inoculated with *P*. *gingivalis* was 654±12 mg HA/cm^3^ (n = 6) whereas that of untreated mice was 753±11 mg HA/cm^3^ (n = 6). Moreover, the loss of bone was so dramatic in the knee joints of B6.DR4 mice that they were clearly evident by simple visual inspection of 3D μCT reconstructions (Fig 6) but are best visualized in 3D animations of these reconstructions (S3–S8 Figs).

## Discussion

In the work of Sato et al. [12], the authors note that inoculation of mice with oral *P*. *gingivalis* did not result in the elicitation of ACPAs. While their results clearly demonstrate a modest exacerbation of arthritis as a result of this treatment, ACPA levels were at those seen in sham-treated animals (eg absent). These results are consistent with our findings for DBA/1 mice inoculated with *P*. *gingivalis*, in the serum of severely arthritic CIA mice of this strain (not shown) as well as that seen for WT B6 mice under either condition. Neither DBA/1 (I-A^q^) nor C57BL/6 (I-A^b^) appear to have the appropriate class II to be able to generate antibodies reactive to the citrullination that is clearly present as a result of challenge with this oral pathogen. In our transgenic B6.DR1 mice, the human RA susceptibility allele HLA-DR1β can fulfill this role, apparently providing sufficient binding and presentation of citrullinated neo-epitopes to drive production of ACPAs. These neo-epitopes can be produced by endogenous murine PAD as a result of inflammatory activation in CIA or by the PPAD generated by *P*. *gingivalis*, citrullinating terminal arginine residues that have been newly generated through the action of gingipains.

One of the more perplexing questions in this field is that concerning the role (if any) of the ACPA in the arthritis pathology development and how the SE influences this. ACPAs have been hypothesized to function through the formation of immune complexes (IC) between ACPA and their citrullinated ligand, and a set of fine specificities for these ligands has recently been reported for SE-bearing RA patients [13]. But how do we make the leap from IC formation to exacerbation of disease, and how does this relate to smoking and the SE? Immune complexes, whether they consist of antibodies of irrelevant provenance [14] or those directed at citrullinated epitopes (ACPAs) can bind to FcRs such as FcγRIII and stimulate the upregulation of pro-inflammatory mediators that are already present in the ongoing (auto)immune response. This hypothesis then suggests that anything that can lead to the generation of additional IC can exacerbate an established immune response. This would cover both periodontal disease and smoking, the former as a result of pPAD activity in the oral cavity and the latter being generated through the activation of endogenous mammalian lung epithelial PAD enzymes and the generation of ACPAs reactive to the resultant citrullinated lung autoantigens.

Another important issue to dissect is that of the nature of the specific epitopes that we analyzed in this study. While the exact nature of the CCP2 ligands is proprietary, CCP2 targets bound by B6.DR1 ACPAs are the very same ones used in the diagnosis of RA. Confusing the issue is the increasing adoption of commercial mouse ACPA detection kits such as those supplied by Bio-Assay Technology Laboratory. These ELISA-based kits can and do detect mouse antibodies to citrullinated epitopes that are generated through the immunization of mice with various synthetic citrullinated moieties. However, unlike the CCP2-based human diagnostic ELISA, the rigor in developing the specific panel of epitopes toward which antibodies are directed has not been applied. It is difficult to understand the relevance of the ACPAs to autoimmune arthritis or to periodontal disease as measured in rodents as the targets were developed through the immunization of mice with synthetic citrullinated ligands. Considering the ACPA measurements we report in this study, while we have not determined the nature of the epitopes, we can at least take some encouragement from the fact that serum from mice generated under two different conditions; either inoculation with *P*. *gingivalis* or as a result of autoimmune arthritis- both yield ACPAs as measured by CCP2 ELISA; the same diagnostic used in rheumatology and periodontology clinics.

ACPA-driven IC formation must not be the only mechanism by which *P*. *gingivalis* exacerbates arthritis because DBA/1 mice (which do not make significant titers of CCP2 ACPAs) also demonstrate a significant exacerbation of collagen-induced arthritis when challenged with oral *P*. *gingivalis* [12, 15]. There remains the possibility that DBA/1 mice do indeed make ACPAs under these conditions, but that the range of epitopes produced does not intersect with those in CCP2 diagnostics. It is also important to remember that IC formation driving enhanced pro-inflammatory responses can exacerbate any chronic inflammatory disease process.

One additional point in this study that bears mention is the identification of a pair of data points from a single mouse in which we detected high levels of ACPAs in a mouse prior to the onset of arthritis. We hypothesize that this may have been an indication that while clinical symptoms of arthritis had not yet developed in this mouse, its appearance was imminent. If our supposition turns out to be accurate, it would be important for the murine CIA model in that one of the difficulties encountered by those wishing to study the effectiveness of anti-inflammatory compounds or disease-modifying drugs is the question of timing. One would like to begin treatment before the appearance of acute inflammation while still allowing the immune response to generate all of the necessary conditions to provide that response. If a spike in ACPA levels can be used to more accurately predict the onset of clinical symptoms, it will give research teams the ability to time their treatments such that they have the best chance of success. Because we know that elevated ACPA levels can indeed be detected in patients prior to the onset of RA, it would provide an important parallel that gives the study greater clinical significance.

## Supporting information

S1 Fig Standard curve used to calculate levels of antibodies to *P. gingivalis*. Affinity purified antibody isolated from pooled hyperimmune serum of mice inoculated with *P*. *gingivalis* was used in a serial dilution on plates coated with sonic extracts of *P*. *gingivalis* strain W83 as outlined in Materials and methods. Example standard curve used for illustrative purposes.
(PDF)

S2 Fig Comparison of Th17 responses to inoculation with *P. gingivalis* vs vehicle (CMC) in WT (B6) or B6.DR1 mice. Following six inoculations with either *P*. *gingivalis* or the carrier vehicle (carboxymethyl cellulose; CMC) we found similar measurable responses to CMC inoculation in both strains but it was lower than that of the B6.DR1 mice inoculated with *P*. *gingivalis*. These data are provided as a control to demonstrate that the elevated responses to *P*. *gingivalis* by the B6.DR1 mice could be significant differences related to the presence of the shared epitope in the B6.DR1 mice.
(PDF)

S3 Fig Animation of 360˚ rotation of knee demonstrating *P. gingivalis*-mediated bone loss. Bone loss can be seen in three-dimensional animation of μCT reconstructions of the left knee joint from a DR4-bearing mouse treated with *P*. *gingivalis* (joint depicted in the upper left of Fig 6).
(MOV)

S4 Fig Animation of 360˚ rotation of knee demonstrating *P. gingivalis*-mediated bone loss. Bone loss can be seen in three-dimensional animation of μCT reconstructions of the right knee joint from a DR4-bearing mouse treated with *P*. *gingivalis* (joint depicted in the upper right of Fig 6).
(MOV)

S5 Fig Animation of 360˚ rotation of knee from untreated mouse. Normal bone structure can be seen in three-dimensional animation of μCT reconstructions of the left knee joint from an untreated DR4-bearing mouse (joint depicted in the lower left of Fig 6).
(MOV)

S6 Fig Animation of 360˚ rotation of knee from untreated mouse. Normal bone structure can be seen in three-dimensional animation of μCT reconstructions of the left knee joint from an untreated DR4-bearing mouse.
(MOV)

S7 Fig Animation of 360˚ rotation of knee from untreated mouse. Normal bone structure can be seen in three-dimensional animation of μCT reconstructions of the right knee joint from an untreated DR4-bearing mouse.
(MOV)

S8 Fig Animation of 360˚ rotation of knee demonstrating *P. gingivalis*-mediated bone loss. Bone loss can be seen in three-dimensional animation of μCT reconstructions of the right knee joint from a DR4-bearing mouse treated with *P*. *gingivalis*.
(MOV)
