# Correction: Influence of the shared epitope on the elicitation of experimental autoimmune arthritis biomarkers

**DOI:** 10.1371/journal.pone.0263754

**Published:** 2022-02-02

**Authors:** Anastasios Karydis, Indra Sandal, Jiwen Luo, Amanda Prislovsky, Amanda Gamboa, Edward F. Rosloniec, David D. Brand

S3 and S6 Figs were mistakenly swapped, as were S4 and S7 Figs respectively. The correctly ordered files can be viewed below.

## Supporting information

S3 Fig Animation of 360° rotation of knee demonstrating *P*. *gingivalis*-mediated bone loss. Bone loss can be seen in three-dimensional animation of μCT reconstructions of the left knee joint from a DR4-bearing mouse treated with *P*. *gingivalis* (joint depicted in the upper left of Fig 6).
(MOV)

S4 Fig Animation of 360° rotation of knee demonstrating *P*. *gingivalis*-mediated bone loss. Bone loss can be seen in three-dimensional animation of μCT reconstructions of the right knee joint from a DR4-bearing mouse treated with *P*. *gingivalis* (joint depicted in the upper right of Fig 6).
(MOV)

S6 Fig Animation of 360° rotation of knee from untreated mouse. Normal bone structure can be seen in three-dimensional animation of μCT reconstructions of the left knee joint from an untreated DR4-bearing mouse.
(MOV)

S7 Fig Animation of 360° rotation of knee from untreated mouse. Normal bone structure can be seen in three-dimensional animation of μCT reconstructions of the right knee joint from an untreated DR4-bearing mouse.
(MOV)
