# Supplementary figures and images for: Influence of the shared epitope on the elicitation of experimental autoimmune arthritis biomarkers
Source: PLoS One. 2021 Apr 15;16(4):e0250177. doi: 10.1371/journal.pone.0250177 (PMC8049293; doi:10.1371/journal.pone.0250177)

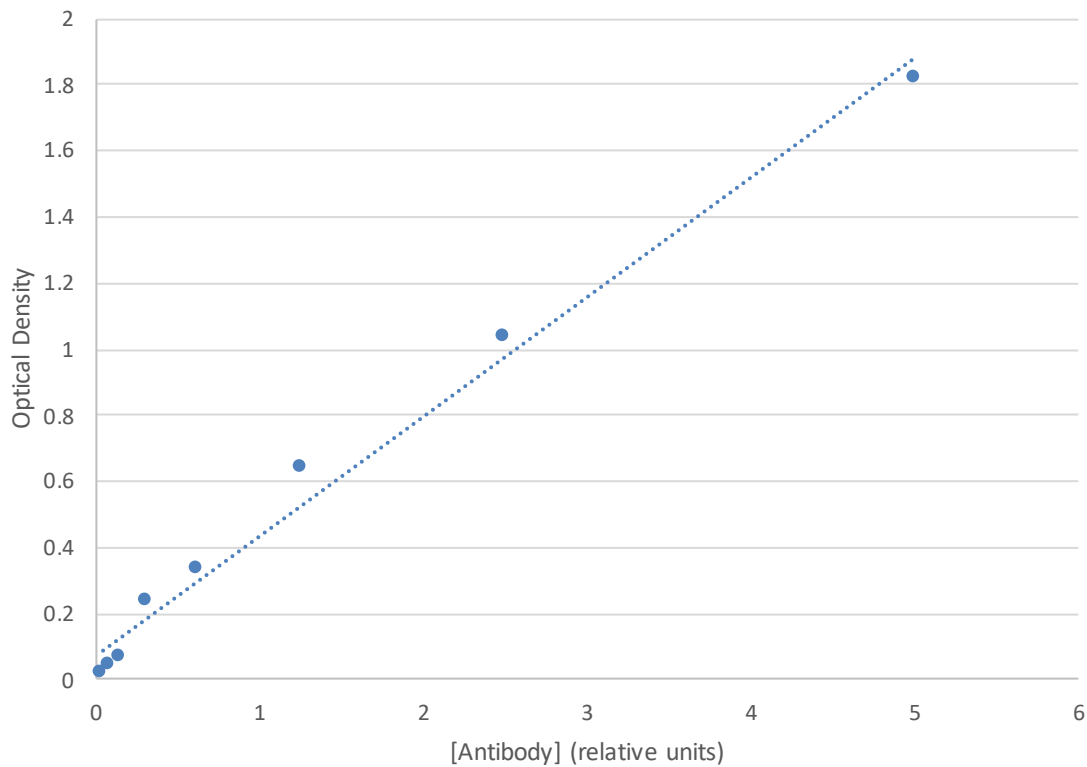

Supplement: S1 Fig — Affinity purified antibody isolated from pooled hyperimmune serum of mice inoculated with P. gingivalis was used in a serial dilution on plates coated with sonic extracts of P. gingivalis strain W83 as outlined in Materials and methods. Example standard curve used for illustrative purposes. (PDF) [file pone.0250177.s001.pdf]

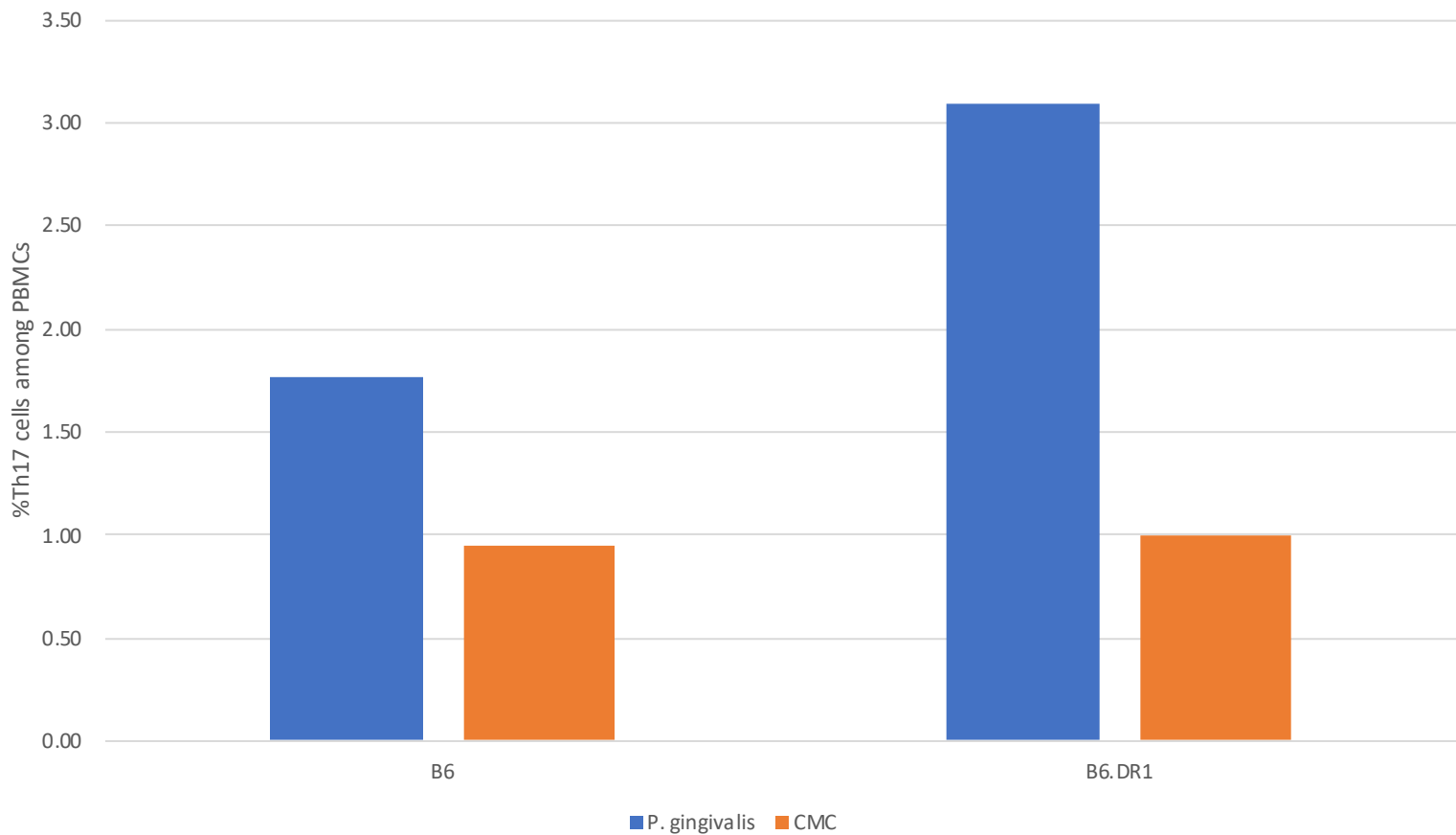

Supplement: S2 Fig — Following six inoculations with either P. gingivalis or the carrier vehicle (carboxymethyl cellulose; CMC) we found similar measurable responses to CMC inoculation in both strains but it was lower than that of the B6.DR1 mice inoculated with P. gingivalis. These data are provided as a control to demonstrate that the elevated responses to P. gingivalis by the B6.DR1 mice could be significant differences related to the presence of the shared epitope in the B6.DR1 mice. (PDF) [file pone.0250177.s002.pdf]
